# Making the best of research investment in pathogens control through biocontrol. How is research correlated with agricultural microbial biological control product availability?

**DOI:** 10.1371/journal.ppat.1011071

**Published:** 2023-01-26

**Authors:** Florența-Elena Helepciuc, Arpad Todor

**Affiliations:** 1 Department of Developmental Biology, Institute of Biology Bucharest, Romanian Academy, Bucharest, Romania; 2 Faculty of Political Science, National University of Political Studies and Public Administration, Bucharest, Romania; Shanghai Center for Plant Stress Biology, CHINA

## Abstract

While using microbial biological control products (MBCPs) to limit pathogens is one of the alternatives to the ecologically unsustainable use of synthetic pesticides that received attention, the last 2 decades have not brought the foreseen leap in developing systematic alternatives based on low-risk plant protection products (PPPs) across the globe. To explain this limited progress, we map the evolution of research on the most successful microbial biological control agents (MBCAs) worldwide. We also map the financing structure in the top funding countries and the European Union (EU) to discern the relevant trends. Available data for the European Union Member States allowed us to discover a country-level and EU-level correlation between strain-level research and biocontrol products’ approval based on those strains.

## Why is the research–microbial biological biocontrol product (MBCP) development nexus important?

In controlling plant pathogens [1], compared to synthetic pesticides, biological control agents (BCAs) have multiple advantages, like the positive impact on yields, and the health of crops, minimal adverse effects on farmworkers’ and communities’ health, the absence of harvesting pause or re-entry period characteristic of chemical pesticides use, and positive public perception [2]. The biocontrol industry has grown in the last decades but reached a worldwide market of only US$1.5 billion [3] and 3.6 billion euros in 2022, of which 800 million euros in the European Union (EU) [4]. It is projected to increase to US$7.5 billion by 2025 [5] but will still represent only 2% of the synthetic pesticides market. Unlike invertebrate BCAs, which generally need no registration and approval, employing microbial biological control agents (MBCA) is highly controlled in developed countries. Despite aiming to promote low-risk alternatives [6], the EU has a more costly and time-consuming double-stage procedure (microbial strain approval at the EU level and product authorization at the national level) [7] than the United States, similar to chemical pesticides. The situation improved after 2018 when some MBCA were labeled low-risk substances [8], the European Food Safety Authority published several pesticide risk assessments to accelerate developments and decrease barriers, and MBCA approval accelerated [9,10]. Nevertheless, factors like the cost of patents, high specificity of the products, costs of product registration, complex application of MBCP, more elaborate production and storage processes [11], the difficulties in identifying efficient strains, and the complex development process leading to efficient microbial biological control products (MBCP), still hamper the development of the sector. Consequently, the fundamental and applied research in biocontrol is one of the most significant ways the scientific community can contribute to agriculture’s sustainability and decrease the time between the appearance of new diseases and the development of new MBCP [12].

## How to evaluate the link?

Studying this relationship is easier said than done, as the limited data availability imposes severe limitations. We have employed Claryvate Analytics Web of Science (WoS) with subcollections to map research and funding evolution. It rigorously identifies the country of the author’s institutions and the funding agency. WoS allows complex search syntax for labeling articles on biocontrol. WoS’s disadvantage is that only the titles, keywords, and abstracts are introduced, not the article’s whole body. As only the species used in experiments are mentioned in the abstract but not the strains, only species-level labeling of the article is possible.

As WoS does not allow strain-level search, we have employed Google Academic (GA), the most exhaustive database containing many articles’ content. Nevertheless, GA is less rigorous, does not have a unique identification number for articles, and cannot differentiate between authors’ countries or funding agencies.

## How fragmented is funding, and what country’s agencies dominate funding?

First, we aimed to establish to what extent the country and financing agencies of biocontrol research have changed in the last 2 decades. We treat the EU as a region because, unlike the other major markets with a one-step registration process, the EU has 2 stages of the procedure. Furthermore, EU MS can access funding for research through European funds. In a recent article, we used WoS data to show that 2007 marked a turning point in the EU’s and China’s approach to biocontrol, as the number of articles exceeded those published by US-based scientists [10]. Here, we investigate if this change in publications is linked to the financing structure of MBCA research.

Data on funding sources for biocontrol articles in WoS between 2005 and 2018 shows that the research is highly fragmented, as 73.4% of grant institutions funded only 1 article, 11.7% 2, and 5.8% 3 articles. Just 1.4% (15 in total) of granting institutions supported more than 6 articles in biocontrol. Out of the 1,077 agencies identified as financing biocontrol-related research published in WoS-indexed articles, the highest number of programs (around 182) and 7 out of the 12 top are Chinese. EU grants are coming only in fifth place, while the USA’s Department of Agriculture (6th place), Department of Health and Human Services (9th place), and National Science Foundation (11th place) are trailing. Not unexpectedly, the national and multinational corporations have rarely financed published research in biocontrol (Bayer– 14 mentions, Novartis– 10 mentions, Merk– 10 mentions, BASF– 1 mention, Monsanto, Syngenta, Koppert–no mention). Results underline the fragmentation of biocontrol research funding. Fragmentation prevents the continuous funding of research through the bioproduct cycle from strain identification to production and limits identifying common research priorities [13].

## Is research correlated with product authorization?

**Fig 1:**
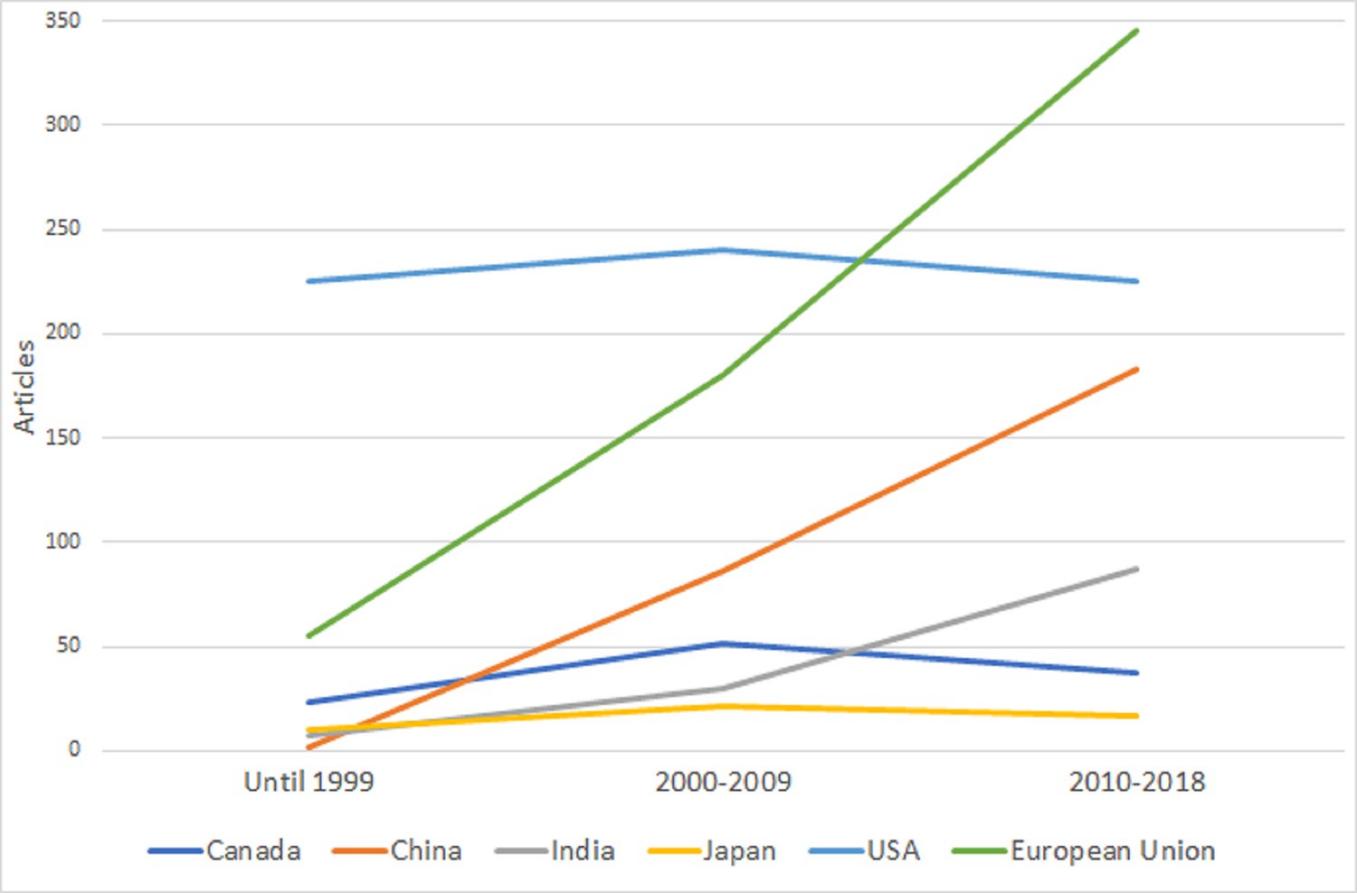
Evolution of published articles in the field of biocontrol using *Bacillus thuringiensis* strains. The vertical axis represents the number of articles published in each of the 3 periods analyzed (until 1999, 2000–2009, and 2010–2018) by country (Canada, China, Japan, and the USA) or regulatory region (European Union).

**Fig 2:**
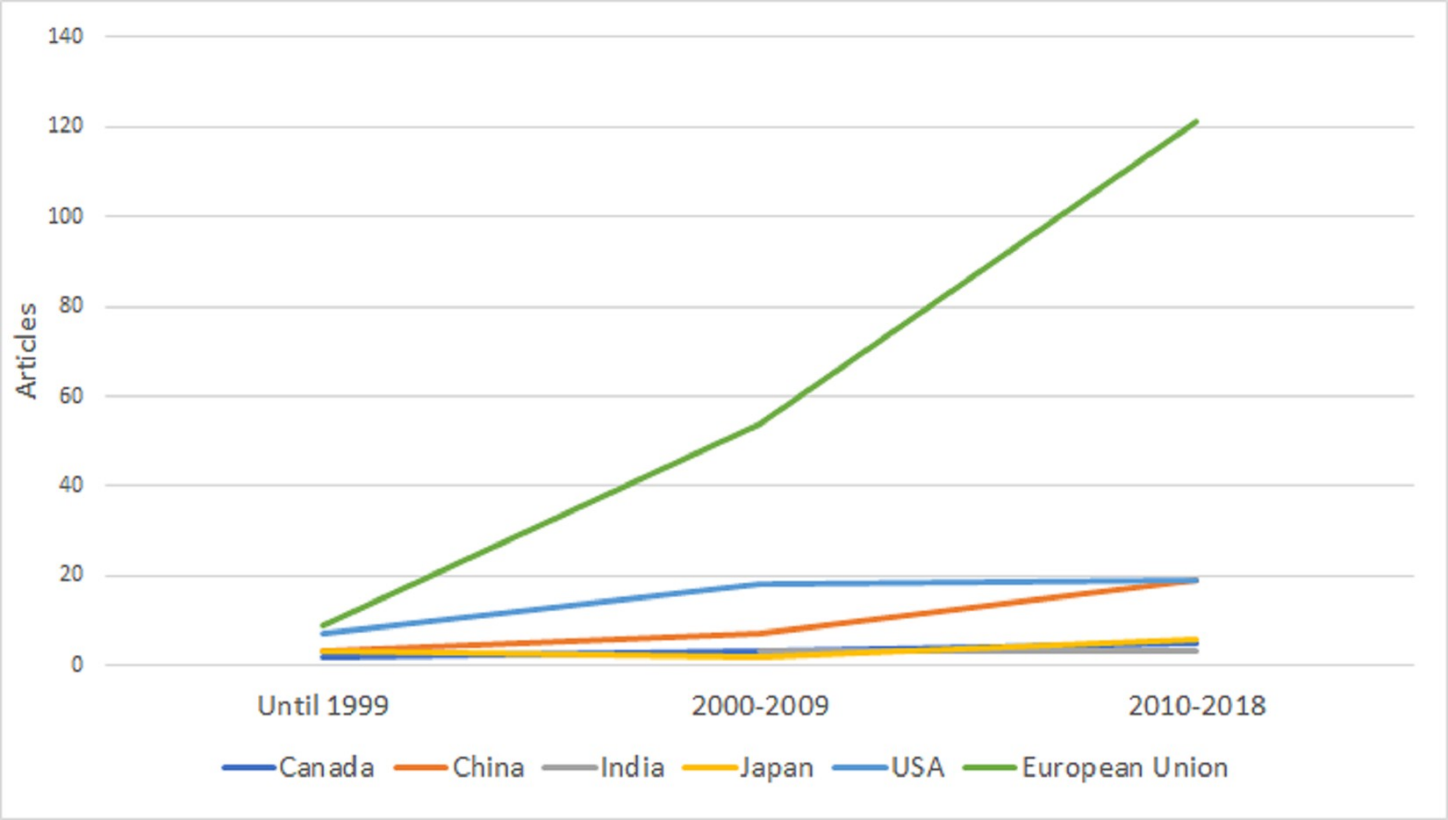
Evolution of published articles in the field of biocontrol using *Aureobasidium pullulans* strains by country. The vertical axis represents the number of articles published in each of the 3 periods analyzed (until 1999, 2000–2009, and 2010–2018) by country (Canada, China, Japan, and the USA) or regulatory region (European Union).

**Fig 3:**
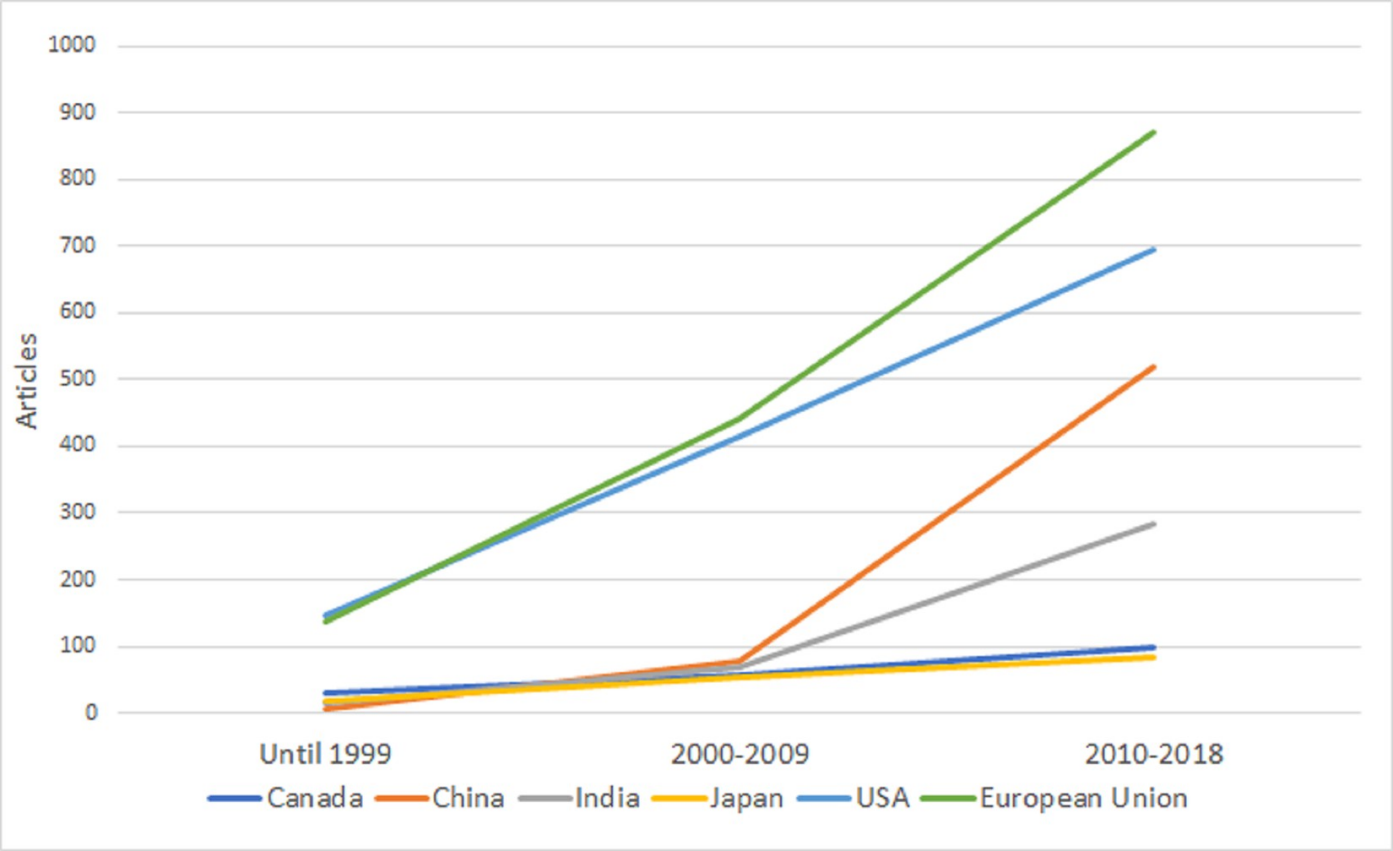
Evolution of published articles in the field of biocontrol using *Bacillus amyloliquefaciens* strains. The vertical axis represents the number of articles published in each of the 3 periods analyzed (until 1999, 2000–2009, and 2010–2018) by country (Canada, China, Japan, and the USA) or regulatory region (European Union).

**Fig 4:**
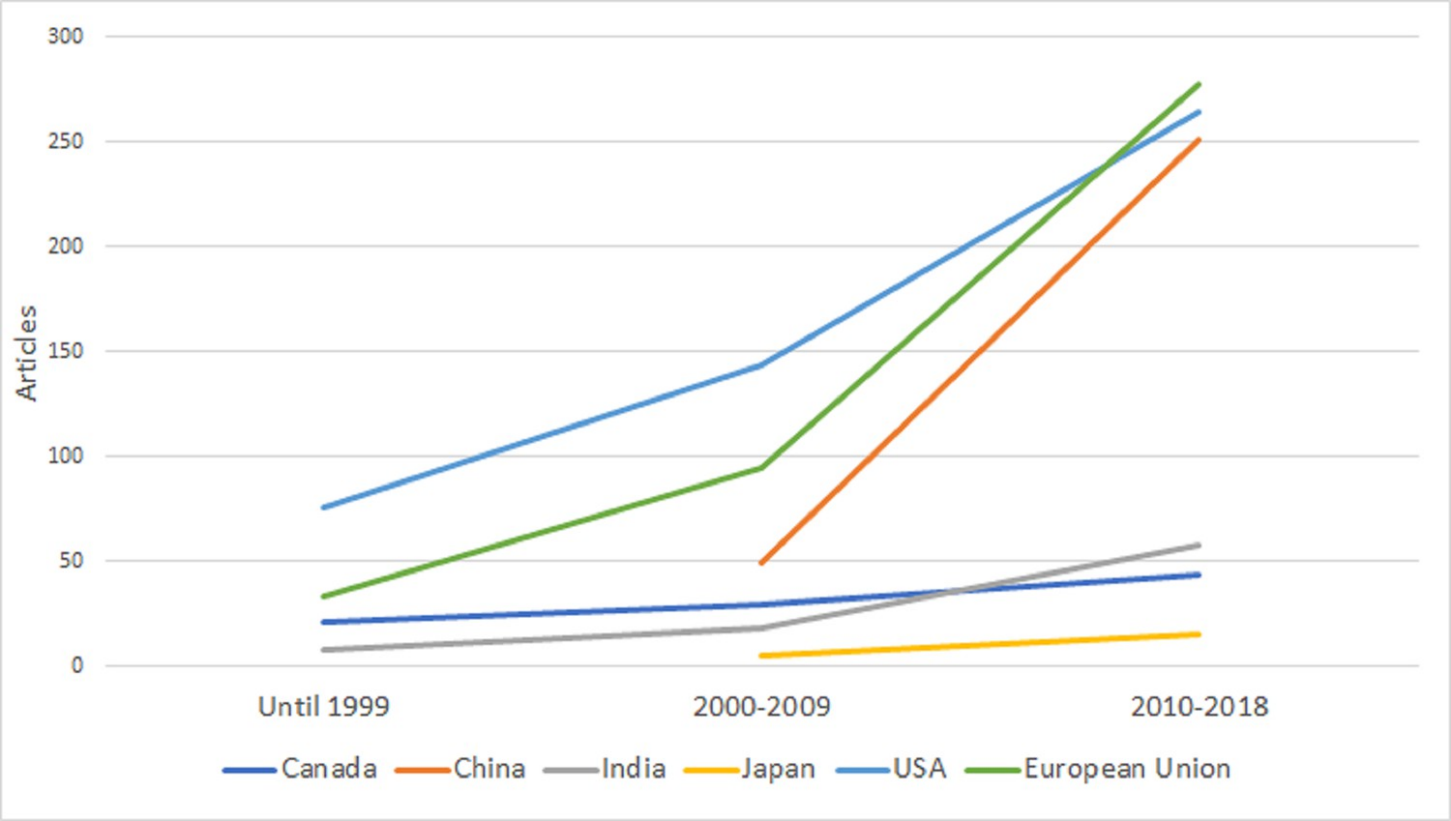
Evolution of published articles in the field of biocontrol using *Beauveria bassiana* strains. The vertical axis represents the number of articles published in each of the 3 periods analyzed (until 1999, 2000–2009, and 2010–2018) by country (Canada, China, Japan, and the USA) or regulatory region (European Union).

Second, we studied if country-level biocontrol research on specific microbial species is correlated with country-level MBCP authorization based on the same species in the EU. We focused only on the 28 EU Member States (MS), as we could collect strain-level data for bivariate statistics using data from GA and the EU Pesticides database [14]. In Figs 1–4, we plot the evolution of research on the 4 species that comprise most of the approved strains by 2019: *Bacillus thuringiensis*, *Aureobasidium pullulans*, *Bacillus amyloliquefaciens*, and *Beauveria bassiana*. Strain-level research increased significantly in all 4 cases in 2010 to 2018, as the EU topped the USA and China. Table 1 shows the bivariate correlations of country-level products’ authorization based on strains from the 4 species (by 2014 and by 2019), and the number of articles on biocontrol in that species (2010 to 2018) is positive. The correlation’s strength increased from 2014 to 2019 for all the species, indicating an increasing link between research and product authorization in the EU MS at the species level.

**Table 1:** The correlation between species with approved strains for biocontrol products in the EU MS and the number of biocontrol articles published between 2010 and 2018 on those species.

	*Aureobasidium pullulans*		*Bacillus amyloliquefaciens* (formerly B. subtilis)		*Beauveria bassiana*		*B*. *thuringiensis*	
	Strains approved by 2019	Strains approved by 2014	Strains approved by 2019	Strains approved by 2014	Strains approved by 2019	Strains approved by 2014	Strains approved by 2019	Strains approved by 2014
Number of articles published 2010–2018	**0.31	**0.25	**0.33	**0.32	**0.40	*0.26	**0.57	**0.55

** Significant at the 0.01 level.

EU, European Union; MS, Member States.

## Does research focus on strains already proven successfully useful for pathogens control?

Third, we increased our evaluation’s resolution by testing the correlation between biocontrol research at the strain level (number of articles from 2010 to 2020) and the number of countries where products based on that strain were authorized. Testing the correlation was possible only for *Bacillus thuringiensis*, *Bacillus amyloliquefaciens*, and *Beauveria bassiana*. Data in the last column in Table 2 shows that the correlation is very high in all 3 cases. It indicates that at this level, too, basic research is more focused on those strains that are already proven successful for pathogen control, a highly counter-intuitive finding. We also highlighted (with bold) if the year a strain is approved in the EU leads to increased articles published on those microbial strains (we included data for some other 4 species). A clear pattern emerges: strain-level research accelerates after approval at the EU level (first stage). This finding indicates that the potential to be fastly used as a strain in biopesticides increases researchers’ interest and the possibility of attracting funding.

**Table 2:** Registered microbial biological control agents in Canada (CA), the European Union (EU), and the United States of America (USA).

							Published articles by period and year														Correlation between the total no. of strains approved in EU countries and the number of articles	
Microorganism	Type of organism	Total EU countries with approved strain in 2019	First EU registration	First US registration	First CA registration	Total articles	Before 2000	2000–2009	2010–2020	2010	2011	2012	2013	2014	2015	2016	2017	2018	2019	2020	2000–2020	2010–2020
*Bacillus thuringiensis* EG 2348	B	23	2009			75	2	**6**	67	3	7	3	5	4	7	6	5	9	9	9	0.45**	0.45**
*Bacillus thuringiensis* SA-11	B	22	2009			71	6	**4**	61	4	5	3	5	3	8	10	7	3	11	2		
*Bacillus thuringiensis* SA-12	B	22	2009			56	6	**3**	47	3	3	4	4	5	6	3	3	6	6	4		
*Bacillus thuringiensis* ssp. *A*izawai GC-91	B	17	2009			4	0	**0**	4			1		1				1	1			
*Bacillus thuringiensis* ssp. *Aizawai* ABTS-1857	B	17	2009	2006		4	0	**0**	4		1	1		1				1				
*Bacillus thuringiensis* ssp. *israeliensis* (serotype H-14) AM65-52	B	6	2009	2006	1989	12	0	**0**	12			2	1	2	1		2	2	1	1		
*Bacillus thuringiensis* ssp. *Kurstaki* ABTS 351	B	24	2009	2010		15	0	**0**	15			1	1		2			6	4	1		
*Bacillus thuringiensis* ssp. *Kurstaki* PB 54	B	24	2009			4	0	**0**	4			1						1	2			
*Bacillus thuringiensis* ssp. *tenebrionis* NB 176	B	7	2008		2001	6	0	**0**	6		1	1					1	1		2		
*Aureobasidium pullulans* DSM 14940 and DSM 14941–2 strains	Y	11	2014	2012	2012	85	0	0	85	1	2	2	7	**4**	7	7	7	14	22	12		
*Bacillus amyloliquefaciens* (formerly *B*. *subtilis*) MBI 600	B	10	2016	1998	2007	193	6	46	141	5	13	7	12	10	16	**17**	11	19	25	6	1.00**	0.95**
*Bacillus amyloliquefaciens IT-45*	B	0	2020			46	0	0	46	0	0	2	1	1	8	5	3	12	12	2		
*Bacillus amyloliquefaciens* AH2	B	0	2020			46	1	9	36	0	1	2	3	5	5	1	4	10	4	**1**		
*Bacillus myloliquefaciens* ssp. *plantarum* (syn. *Bacillus subtilis* var. *amyloliquefaciens*) D747	B	8	2014	2011	2015	165	0	2	163	0	2	12	3	6	20	12	18	31	40	**19**		
*Beauveria bassiana* ATCC 74040	F	16	2009	1995		309	8	**47**	254	6	12	14	26	24	23	25	28	47	31	18	0.81**	0.83**
*Beauveria bassiana* GHA	F	16	2009	1995	2009	1042	49	**229**	764	47	63	56	73	60	64	71	93	95	85	57		
*Beauveria bassiana* IMI389521	F	0	2019			11	0	0	11					1	1	3	1	2	**3**			
*Beauveria bassiana* NPP111B005	F	2	2017			7	0	0	7		1		1		1		**0**	2	1	1		
*Beauveria bassiana* PPRI 5339	F	0	2019	2018	2018	30	1	2	27	1			1		1	1	1	6	**10**	6		
*Bacillus firmus i-1582/ Bacillus firmus I-201582–2 strains*	B	8	2013	2008	2012	173	2	6	165	1	4	5	10	12	11	14	17	34	**35**	22		
*Bacillus pumilus QST 2808*	B	0	2014	1990		141	0	5	136	4	5	4	11	16	17	15	14	21	**17**	12		
*Bacillus subtilis QST 713*	B	22	2007	1991		78	0	0	78	4	5	6	8	4	3	7	3	9	**13**	16		
*Ampelomyces quisqualis AQ10*	F	12	2004	1994		382	8	72	302	26	33	34	39	25	27	35	22	16	**28**	17		

** Significant at the 0.01 level.

## Conclusions

Data shows a clear intensifying of biocontrol-related research, especially in the EU and China, compared with the US, after 2006. However, financing for this research is very fragmented across different agencies and programs. Only China has a consistent set of larger financing agencies. The US is trailing in total publications on biocontrol, research on the most successfully used in biocontrol microbial strains, and financing.

For the species that generated the highest interest in the number of authorized MBCP in the EU MS, the increase in research intensity is evident compared with other countries investigated in approved strains and the number of countries approving products. Furthermore, strain-level research also appears to be correlated with the success of MBCPs authorization based on those strains.

The positive correlation between increased biocontrol research and MBCP authorization could provide a virtuous circle that could sustain an exponential increase of MBCP availability in the next decade, products that, in combination with improved recent legislation [15], the introduction of Integrated Pest Management (IPM) and organic agriculture, could set the path for a more sustainable and environmentally friendly effort to control pathogens. The fragmentation pattern in financing biocontrol research in the EU and the USA probably prevents identifying some priorities. It also prevents faster sampling cycles, strain isolation, and selection, patenting, assessment, storage and formulation, and product registering.
